# Family Function and Child Adjustment Difficulties in the COVID-19 Pandemic: An International Study

**DOI:** 10.3390/ijerph182111136

**Published:** 2021-10-23

**Authors:** Sarah Foley, Farzaneh Badinlou, Karin C. Brocki, Matilda A. Frick, Luca Ronchi, Claire Hughes

**Affiliations:** 1 Moray House School of Education and Sport, University of Edinburgh, Edinburgh EH8 8AQ, UK; 2 Department of Psychology, Uppsala University, 751 42 Uppsala, Sweden; farzaneh.badinlou@psyk.uu.se (F.B.); Karin.Brocki@psyk.uu.se (K.C.B.); matilda.frick@psyk.uu.se (M.A.F.); 3 Centre for Psychiatry Research, Department of Clinical Neuroscience, Karolinska Institutet, 117 63 Stockholm, Sweden; 4 Department of Brain and Behavioural Sciences, University of Pavia, 27100 Pavia, Italy; luca.ronchi@unipv.it; 5 Centre for Family Research, University of Cambridge, Cambridge CB2 3RQ, UK; ch288@cam.ac.uk

**Keywords:** COVID-19, child adjustment, family, risk, distress, international

## Abstract

To estimate specific proximal and distal effects of COVID-19-related restrictions on families on children’s adjustment problems, we conducted a six-site international study. In total, 2516 parents from Australia, China, Italy, Sweden, the United Kingdom, and the United States of America living with a young child (*M_age_* = 5.77, *SD* = 1.10, range = 3 to 8 years, 47.9% female) completed an online survey between April and July 2020. The survey included the Strengths and Difficulties Questionnaire and family risk factors (parent distress, parent–child conflict, couple conflict, and household chaos) as well as a scale to index COVID-19-related family disruption. Our analyses also included public data on the stringency of national restrictions. Across the six sites, parental responses indicated elevated levels of hyperactivity, conduct, and emotion problems in children from families characterized by heightened levels of parent distress, parent–child conflict, and household chaos. In contrast, increased peer problems were more strongly related to COVID-19-related social disruption and stringency measures. Mediation models demonstrated that associations between COVID-19 social disruption and child difficulties could be explained by parental distress. Taken together, these results suggest that although the experience of the pandemic differed across countries, associations between COVID-19-related family experiences and child adjustment difficulties were similar in their nature and magnitude across six different contexts. Programs to support family resilience could help buffer the impact of the pandemic for two generations.

## 1. Introduction

While the COVID-19 pandemic has dramatically restricted everyone’s lives, its impact on young children may be especially important for at least four reasons. First, according to developmental systems theory [1], children’s trajectories and developmental outcomes are the result of dynamic interactions between individual (e.g., genetic, neural, biological, and cognitive), family, and contextual factors. Crucially, children’s outcomes show heightened susceptibility to the influence of family processes, especially during sensitive periods of development. One such period is the preschool to early school-age years, when children make the important transition to school and begin to develop friendships [2]. Second, compared with older school-aged children and adolescents, young children spend much of their time near parents and so may be indirectly affected by the impact of the pandemic via parental wellbeing and availability (e.g., [3]). Third, young children lack the experience and skills needed to maintain social relationships via virtual platforms, so their social interactions depend upon face-to-face contact and outdoor play [4]. Fourth, difficulties in understanding health risks may amplify the stress associated with changes and restrictions, for example, “stay-at-home” rules and mass school closures.

### 1.1. Cascading Effects of COVID-19 Experiences on Child Adjustment

During times of stress and crisis, children are likely to require closer contact with caregivers [1,5]. Evidence to support this view comes from reports of increased child clinginess, distractibility, and irritability during the pandemic [6,7]. Compounding these child demands, the pandemic has also generated widespread financial strain, family conflict, and concern for loved ones [8], while mass school closures have obliged many working parents to deliver home-schooling, further escalating parent stress [9]. Parental distress in response to these multiple stressors may have cascading effects on child mental health [10], as illustrated by worrying evidence of a significant increase in child abuse during the pandemic [11]. In presenting this conceptual framework, Prime and colleagues [10] draw on evidence illustrating both direct and indirect adverse effects of natural disasters/economic crashes on parent wellbeing, with negative consequences for children.

To date, research on the impact of the pandemic on young children has typically relied on data from single-site samples, raising questions regarding the generalizability of the study findings. This is significant as developmental systems theory (e.g., [12]) emphasizes the importance of interactions between familial and societal factors (i.e., micro and macro levels of influence) that vary across time and place. Contrasts in both levels of virus transmission and political motivations have led to significant between-country variation in responses to the COVID-19 pandemic (c.f. [13]) such that families have had markedly different pandemic experiences. This between-country variation accentuates within-country socioeconomic and racial inequalities. Focusing on between-country differences, the current study adopted an international design combining data from six different countries to estimate specific effects of COVID-19-related experiences on child adjustment difficulties. Informed by Prime’s theoretical framework [10], we also sought to test whether the effect of COVID-19 disruption on child adjustment difficulties was mediated by parental distress. To this end, given the hypothesized importance of parental wellbeing, a preliminary goal of the current study was to test whether our measure of parent distress was invariant across site.

### 1.2. Cross-Cultural Differences in Child Adjustment during the Pandemic

A survey of almost 18,000 caregivers in 46 countries showed that markers of child distress (e.g., sleep disturbances and aggression) were positively related to the length of school closures [14]. Whilst this suggests a pervasive adverse effect of COVID-19 restrictions on children’s psychosocial adjustment, meaningful cross-cultural research hinges upon establishing the equivalence of measures. This entails testing whether instruments assessing child adjustment difficulties show “measurement invariance” across groups [15]. Adopting this approach, the current international consortium brought together researchers with a common interest in family influences on children’s adjustment across six countries with diverse COVID-19 experiences: Australia, China, Italy, Sweden, the United Kingdom, and the United States of America.

In Spring 2020, these six countries were at different points of virus transmission. Notably, infections in China preceded cases in the other sites. These six sites also adopted distinct policies to limit the spread of the virus. Indeed, Sweden was widely considered an outlier on the global stage with their relatively liberal level of pandemic restrictions and, crucially for young children and their parents, lack of national school closures. After demonstrating across-site measurement invariance for parental SDQ ratings [16], we showed that during the pandemic, Swedish parents reported the lowest levels of child peer problems and the highest level of child prosocial behaviors, whilst British parents reported the highest levels of child emotional and hyperactivity problems. These descriptive data are important, but research is needed to understand the mechanisms underpinning these adverse effects [17]. The overarching aim of the current study was to test whether similar or distinct family and COVID-19 experiences are associated with individual differences in children’s adjustment difficulties across these six different countries.

### 1.3. Domain-Specific Influences on Child Adjustment

Given that the COVID-19 pandemic has had multiple impacts on family life, an obvious question is whether different risk factors show distinct patterns of association with child outcomes. As noted above, the developmental systems framework [1] posits three levels of influence on developmental outcomes: individual, family context, and society/culture. Empirical evidence also highlights the domain-specific nature of influences within any one of these levels. For example, in a longitudinal and detailed observational study, Hughes and Devine [18] found that while some family measures (e.g., home learning environment and frequency and complexity of family talk) have a relatively global influence, other measures (e.g., negative control and parental scaffolding) show specific associations with distinct aspects of children’s development.

Three pandemic studies indicate that child adjustment problems are associated with poorer parent mental health, household chaos, and family conflict. However, these family risk factors have yet to be considered in tandem or across different contexts. As a result, little is known about either their relative salience and independence or about the generalizability of pathways. Addressing these gaps, the current international study examined the unique impact of parent mental health, household chaos, and family conflict on both child internalizing and externalizing problems in the early stages of the pandemic. Findings from two studies suggest a differentiated pattern of associations between family risk factors and child outcomes in the context of COVID-19. First, consistent with robust evidence for a negative impact of parental depression on children’s behavioral and emotional problems [19,20], Romero et al. [21] surveyed parents of 1049 Spanish 3- to 12-year-olds and found that parents’ self-reported distress showed moderate links with ratings of child externalizing problems but was unrelated to ratings of children’s prosociality. Second, in an American study of 303 parents of 6-year-olds, Waller et al. [22] reported that reduced parental warmth and increased harsh discipline during the pandemic were each associated with elevated levels of callous unemotional traits, but only harsh parenting was associated with elevated levels of conduct problems. These findings are consistent with theoretical accounts attesting to the negative impact of “coercive cycles” of conflictual parent–child interactions (rather than simply the absence of warmth) on children’s conduct problems (e.g., [23]). By contrast, less differentiated findings have emerged from a socially diverse American sample of preschoolers [24]. Specifically, increased levels of household chaos and parenting stress appear associated with elevated depressive symptoms and externalizing problems [24]. These domain-general findings are consistent with longitudinal work with 511 families establishing the negative impact of household instability and disorganization on a variety of outcomes across China, Colombia, Jordan, Kenya, the Philippines, and Thailand [25].

Within the growing body of family studies of child adjustment in the context of the pandemic, the potential role of couple relationship quality has as yet received very little attention. This is surprising as parallels between economic adversities and the pandemic indicate that both can affect child wellbeing via couples’ dyadic processes, such as increasing hostility or withdrawing support [26]. Associations between conflict and child negative outcomes may be direct, for example, via social learning mechanisms, triggering child distress or undermining feelings of security [27], or indirect via impacting the quality of parent–child relationship [28]. Thus, in addition to considering parent–child conflict, our study sought to test whether couple relationship conflict also had a deleterious effect on children’s adjustment and, crucially, whether patterns of association between family risk factors and children’s psychosocial problems were similar across six different pandemic contexts.

### 1.4. The Current Study

Our multisite design served two purposes: (i) to test whether the family processes implicated in the impact of the pandemic on children’s psychosocial functioning are similar across the six sites and (ii) to disentangle the impact of proximal (i.e., family disruption) versus distal (i.e., stringency of government restrictions) COVID-19-related experiences on children’s adjustment difficulties. Alongside these cross-cultural comparisons, our study addressed two further questions. First, do family risk factors and COVID-19-related experiences show specific influences on child adjustment difficulties? Here, we hypothesized independent contributions from each risk factor to child difficulties but anticipated that more proximal COVID-19-related disruption would exert a stronger influence than distal factors (i.e., government stringency). We adopted an exploratory approach when considering whether the magnitude of associations would differ by child outcome. Second, does parental distress mediate the association between COVID-19 social disruption and child adjustment difficulties? Here, we hypothesized that COVID-19-related social disruption would indirectly impact child adjustment difficulties via elevated levels of parental distress.

## 2. Methods

### 2.1. Participants and Procedure

Between 1 April and 7 July 2020, we recruited 2516 parents with one or more children aged 3 to 8 years old (*M*_age_ = 5.77, *SD* = 1.10) to participate in an online survey (via Qualtrics) of family wellbeing and child adjustment in the COVID-19 pandemic. To be eligible to participate, parents had to be aged 18 or above, report no major psychiatric problems or learning difficulties, and have one or more child aged 4–6. Parents were asked to choose one child in the relevant age group when completing the survey. Following ethical review in each site (and translation from English), the survey was advertised widely via social media and university/school mailing lists in Australia, China, Italy, Sweden, the United Kingdom (UK), and the United States of America (USA).

In total, 6.4% of respondents were in Australia (*n* = 161), 13.4% were in China (*n* = 336), 9.7% were in Italy (*n* = 244), 31.6% were in Sweden (*n* = 795), 28.1% were in the UK (*n* = 706), and 10.9% were in the USA (*n* = 274). As described in Table 1, respondents were typically female (81.5% female and 8% male, with 10.5% preferring not to say), aged 21 to 65 years old (*M_age_* = 37.34, *SD* = 5.39 years), and highly educated (63.1% undergraduate or higher degree or equivalent vocational qualification). Only 50% reported their ethnicity, of which 50% identified as white, 29% as Asian, and 21% as having mixed or multiple ethnicities. Between-site contrasts for each of these demographic characteristics were weak (see Table 1); however, these subsamples are not considered to be representative of all parents and children in each site, in particular those from low socioeconomic backgrounds.

### 2.2. Measures

#### Child Adjustment

Participants completed the parents’ version of the Strengths and Difficulties Questionnaire (SDQ), which is used globally to screen for children’s social, emotional, and behavioral problems [29]. The SDQ consists of 25 statements, each rated on a three-point scale (not true, somewhat true, and certainly true), with five items yielding a prosociality subscale and 20 items relating to child difficulties, namely hyperactivity and emotional, peer, and conduct problems [29,30]. Multiple group confirmatory factor analysis confirmed that a 5-factor solution was the best fitting model and showed across-site scalar invariance [16]. For the purposes of this study, we focused on the four child difficulties subscales.

### 2.3. COVID-19 Experience

*Social Disruption.* A 5-item composite was created to index whether the COVID-19 pandemic had led to respondents experiencing disruption. The first item asked if the parent had experienced a change in employment (i.e., unemployment) because of the COVID-19 pandemic (0 = no, 1 = yes). The second item asked if the parent had experienced a drop in income due to the COVID-19 pandemic (0 = no, 1 = yes). The last three items asked respondents how much they had experienced financial strain, work/family conflict, and worry about loved ones as a result of the COVID-19 pandemic, with these three items rated on a 4-point scale: “not at all”, “very little”, “moderate”, and “very much”. To index any level of disruption, scores were dichotomized so that respondents who indicated “not at all” received a 0 and those who responded “very little”, “moderate”, or “very much” received a 1. Total scores for social disruption ranged from 0 to 5; higher scores denoted greater disruption.

*Stringency.* We extracted data from the Oxford COVID-19 Government Response Tracker (OxCGRT), which collects twice-weekly data on governmental responses to the COVID-19 pandemic across countries. The composite stringency index combines nine indicators, including government containment and closure policies (e.g., school and workplace closure) and public health campaign messaging (e.g., targeted versus general public health information) [13]. For each of the nine indicators, individual ordinal values are given for the last week, with a half-point subtracted if the score is general rather than targeted. The scores are then rescaled by their maximum value to create a score ranging from 0 to 100. We created individual measures of the macro level constraint experienced by a family by matching the OxCGRT data with the date the respondent completed the questionnaire.

### 2.4. Family Risk Factors

*Parental Distress.* To index parents’ current mental health, we included the widely used General Health Questionnaire-12 (GHQ-12) [31], which has been translated into more than 38 languages such that each site could adopt a previously validated translation. On a 4-point scale, respondents were asked whether they experienced/have experienced a particular symptom or behavior over the past two weeks. The bimodal (0-0-1-1) was used for scoring system (0 = less than usual, 0 = no more than usual, 1 = rather more than usual, and 1 = much more than usual). A total sum score was created, with higher scores indicating greater distress (α = 0.89).

*Parent–Child Conflict.* Using one item on a 5-point scale developed for the purpose of this study, respondents indicated whether they had experienced an increase in conflict with the target child (1 = marked increase, 2 = small increase, 3 = no change, 4 = small decrease, and 5 = marked decrease). Scores were revered so that a high score reflected more conflict.

*Couple Conflict*. Parents’ current relationship quality was measured with 10 items developed for the purpose of this study. Using a 5-point scale (1 = rarely, 2 = sometimes, 3 = in half of our interactions, 4 = often, and 5 = nearly always), respondents indicated the frequency of behaviors that signal relationship problems (e.g., criticism, controlling, and verbal/physical abuse). A mean score was created, with a higher score denoting more conflict between parents (α = 0.95).

*Household Chaos.* Each of the 15 items in the Confusion, Hubbub, and Order Scale (CHAOS) [32] had a 4-point scale enabling respondents to indicate how strongly they agreed with statements about the current level of noise, crowding, and routines in their home (1 = very much like our home, 2 = often like our home, 3 = a bit like our home, and 4 = not at all like our home). A mean score was created, with higher scores indicating a more chaotic household (α = 0.75).

*Demographics.* Parents also provided information on their child’s age and gender as well as indicators of their socioeconomic status (SES). A SES composite was developed comprising measures of the parent(s) education, occupation, and housing. Specifically, using a scale relevant to their own country, respondents reported on their own educational attainment and, if applicable, the educational attainment of the target child’s other primary caregiver. These scores were then recoded to reflect the closest equivalent in the British system (i.e., high school/secondary, post-secondary, undergraduate, or higher degree). Next, respondents reported on their own occupation, which was coded according to the UK Standard Occupation Classification system [33]. Occupations were coded as low-level (e.g., sales and factory work), mid-level (e.g., skilled-trade and services) and high-level (e.g., managers and professionals). Finally, respondents were asked to report on their household, specifically the number of bedrooms in their home and whether their home was “small and cramped”, “small but adequate”, “quite spacious”, or “very spacious”. A z-score was created for each item. The final aggregate reflected a mean of z-scores from four to six items, whereby a high score reflected higher SES (α = 0.67).

### 2.5. Analysis Plan

Analyses were conducted using M*plus* Version 8 [34]. Prior to examining the main research questions, we examined the latent factor structure of ratings of parent distress (GHQ). We evaluated model fit using three primary criteria: comparative fit index (CFI) > 0.90, Tucker–Lewis index (TLI) > 0.90, and Root Mean Square Error of Approximation (RMSEA) < 0.08 [35]. To test measurement invariance across site, we used multigroup CFA, which involves systematically adding equality constraints to the model and testing the change in model fit of these nested models [36]. Nested model comparisons were judged to be invariant if the CFI decreased by ≤0.020 and RMSEA increased by ≤0.003 [37]. From this basis, we then conducted between-site comparisons of our independent variables of interest and examined correlations between the main measures (Table 2).

Next, we adopted a path modeling approach to examine the associations between COVID-19-related experiences, family processes, and child adjustment [34]. We used a maximum likelihood estimator with robust standard errors (MLR) and evaluated model fit using Brown’s [35] recommended criteria. Model parameters and standard errors were estimated in M*plus* using all available data [38]. To assess whether path estimates were similar across countries, a multiple group model was fitted [35]. Brown [33] notes multiple-group solutions can be evaluated when the group sizes vary. To this end, we fitted a model in which all paths were freely estimated across site and subsequently compared this model to a model in which all paths were constrained to be equal across site. We assessed differences in model fit using the Satorra–Bentler χ^2^ difference test [39]. Finally, we tested whether parent distress mediated the association between COVID-19 disruption and child adjustment problems using M*plus*’ bootstrapping procedures (5000 bootstrap samples).

## 3. Results

### 3.1. Preliminary Analyses: Between-Site Differences in Parents’ COVID-19 Experience and Distress

**Figure 1:**
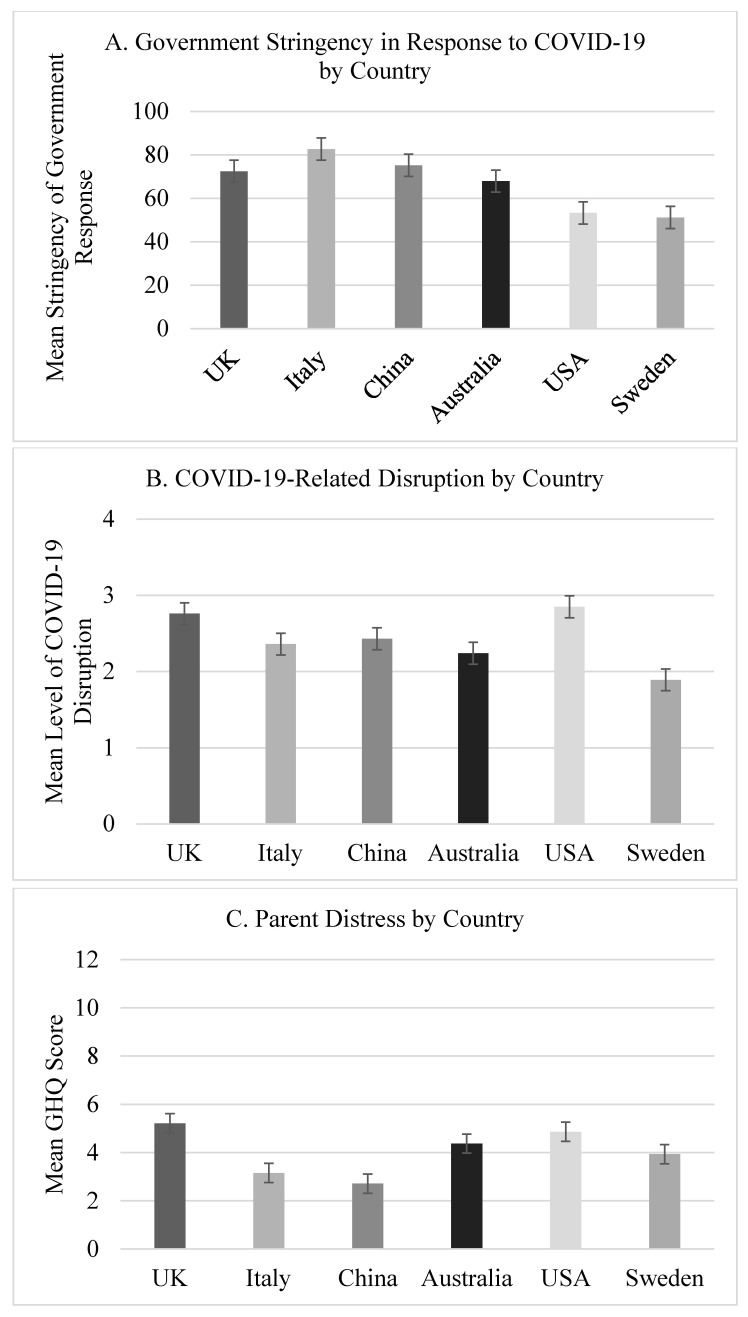
Between-site comparisons of (**A**) government stringency in COVID-19 response between April and July 2020, (**B**) COVID-19-related family disruption, and (**C**) parent distress.

As illustrated in Figure 1, there were between-site contrasts in national government stringency scores (*F*(5) = 755.84, *p* < 0.0001, η^2^ = 0.60) and in the experience of COVID-19-related social disruption (*F*(5) = 38.34, *p* < 0.0001, η^2^ = 0.07). Due to the frequency of planned post-hoc contrasts, we applied a more stringent alpha rate (*p* < 0.003). In terms of stringency scores, there were significant contrasts between most countries, aside from between China and Sweden. For COVID-19-related social disruption, families in the USA experienced a greater level of disruption compared to families in all sites, aside from the UK, while families in Sweden reported less disruption than all sites, except for Australia. Families in the UK reported more disruption than those in Italy, Australia, and Sweden. 

Prior to comparing between-site differences in parental distress, we tested for cross-site measurement invariance of the GHQ using multiple-group confirmatory factor analysis. A partially scalar invariant single-factor model with factor loadings and thresholds free to vary across sites for only one out of 12 items (item 8 “Been able to face up to your problems”) showed a good fit to the data (RMSEA = 0.082 [0.076, 0.087], CFI = 0.969, TLI = 0.967). This suggests that across site, the organization of the construct is similar (i.e., configural invariance), with each item contributing in a similar way to the construct (i.e., metric invariance), and the cut-off underling the distribution of scores are consistent (i.e., scalar invariance). Together, this suggests that the GHQ is appropriate for use across different contexts during the pandemic.

Using this partially scalar invariant GHQ model, we then tested for mean differences in the latent factor for parent distress across sites. Parents in the UK reported significantly more distress than parents in Italy, China, and Sweden, whilst American parents also reported higher levels of distress than Italian or Swedish parents.

### 3.2. COVID-19-Related Experience, Family Risk Factors, and Child Adjustment

We regressed the four child difficulties subscales: conduct problems, hyperactivity, emotion problems, and peer problems onto variables measuring family risk factors (parent distress, parent–child conflict, couple conflict, and household chaos), COVID-19 experiences (disruption and stringency), and demographics (child age, sex, and SES). The model showed good fit (RMSEA = 0.062, 90%CI [0.053, 0.071], CFI = 0.974, TLI = 0.923) and explained a significant proportion of the variance in child hyperactivity (*R*^2^ = 0.29), conduct problems (*R*^2^ = 0.36), emotion problems (*R*^2^ = 0.24), and peer problems (*R*^2^ = 0.16).

As illustrated in Table 3, increased levels of parent distress, parent–child conflict, and household chaos were associated with higher levels of problem behaviors. A Wald chi-square test of model constraints revealed the association between parent distress and child problems was similar in magnitude between conduct, hyperactivity, and emotion problems but weakest for peer problems (*χ*^2^ (3) = 54.38, *p* < 0.0001). There was a stronger association between parent–child conflict and conduct problems than between parent–child conflict and hyperactivity, emotional, or peer problems (*χ*^2^ (3) = 68.81, *p* < 0.0001). The association between household chaos and conduct problems was also stronger than the association between household chaos and emotion or peer problems (*χ*^2^ (3) = 35.63, *p* < 0.0001). There was a stronger association between couple conflict and emotion and conduct problems than with hyperactivity (*χ*^2^ (3) = 39.28, *p* < 0.0001). COVID-19-related disruption and stringency were both associated with greater child difficulties but more strongly associated with peer problems than with the other three dimensions (*χ*^2^ (3) = 12.91, *p* < 0.0001; *χ*^2^ (3) = 23.91, *p* < 0.0001). Specifically, we fitted a multiple-group model in which all paths were freely estimated across site and subsequently compared this model to a model in which all paths were constrained to be equal across site. Compared to the baseline model, this constrained model indicated no significant differences in the pathways between sites (Δχ^2^ = 16.12, *p* > 0.05).

### 3.3. COVID-19-Related Disruption and Child Adjustment: The Mediating Role of Parental Distress

Following this, using bootstrapping procedures [40], we tested whether parental distress mediated the impact of COVID-19-related social disruption on children’s adjustment difficulties. The unstandardized estimate of the indirect effect and 95% confidence intervals with 5000 bootstrap samples was significant for hyperactivity (0.04 [0.03, 0.05], *p* < 0.001), conduct problems (0.03 [0.02, 0.041], *p* < 0.001), and emotion problems (0.04 [0.03, 0.05], *p* < 0.001) but not peer problems (0.01 [0.00, 0.02]).

## 4. Discussion

Despite significant between-site contrasts in mean levels of parental distress and experiences of COVID-19, two key findings highlighted the similarity of associations between family risk factors, COVID-19 experiences, and children’s psychosocial adjustment in the early stages of the COVID-19 pandemic across the six sites. First, children from families characterized by parental distress, increased levels of parent–child conflict, and chaos showed elevated levels of hyperactivity, conduct, and emotion problems. In contrast, increased peer problems were more strongly related to COVID-19-related experiences (both stringency of national restrictions and family-level social disruption). The magnitude of these associations was strongest for chaos, while couple conflict was not significantly related to child adjustment problems. This differentiated pattern of associations supports domain-specific models of social influences on child development and highlights the potential multiplicity of mechanisms implicated in young children’s psychosocial adjustment in the context of the pandemic [41]. Second, our analyses revealed indirect associations between COVID-19-related social disruption and child adjustment difficulties that were mediated by parental distress. Below, we discuss possible explanations for these findings considering our sample limitations.

### 4.1. Family Dysfunction and COVID-19 Experiences Show Direct Links with Child Difficulties

Over and above effects of child age and family SES, increased levels of problems across each of the four problem subscales of the Strengths and Difficulties Questionnaire [29,30] were associated with both micro (parent distress, parent–child conflict, couple conflict, and household chaos) and macro (national stringency) levels of influence. This multiplicity of associations is consistent with developmental systems framework’s central premise that children’s psychosocial development should always be considered in context and that children are particularly responsive to principal relationships within their microsystem [1,12].

While family risk factors were weakly associated with peer problems, they explained around a third of the variance in ratings for hyperactivity, emotion problems, and conduct problems, strengthening the view that coregulation (reflecting parent–child reciprocity, responsiveness, and cooperation) provides a key foundation for behavioral adjustment (e.g., [42]), that is, children’s acquisition of useful skills and habits for regulating their emotions and behavior hinges upon parental warmth and structure, which can be constrained by mental health problems and household chaos [43].

Our findings also mirror reports of a protective effect of family routines on children’s internalizing and externalizing symptoms during the pandemic in the USA [24]. As economic insecurity, irregular employment, and limited access to childcare all predict household chaos (e.g., [44]), elevated levels of family chaos are likely to persist. Given that initial levels and increases in the quality of the home environment predict gains in school readiness [45], a clear practical implication of our results is the need to support parents in their efforts to provide a stable home environment.

Family risk factors exerted a weaker effect on peer problems, which is unsurprising given mass school closures. Instead, highlighting the impact of depriving children of the stimulation and enrichment associated with interactions with other children, peer problems were more clearly associated with the country-wide severity of containment and family-level disruption. On this latter point, it follows that in experiencing unemployment, financial strain, work–family conflict, and worry about loved ones, parents themselves are (or feel) unable to participate in typical group practices, thus limiting children’s opportunities to observe and practice their social skills [46]. Importantly, positive peer relationships are associated with a host of other child outcomes, including the development of children’s identity, socio-cognitive skills, and understanding of social norms [47]. As a result, “catch-up” interventions need to extend beyond the academic domain to include emotional support and enriched opportunities for peer play. Notably, these suggestions are possible due to our inclusion of peer problems as a specific outcome of interest, a domain largely absent from earlier research that demonstrated large-scale economic crashes were linked to decline in children’s mental health [48].

### 4.2. COVID-19-Related Disruption, Family Risk Factors, and Child Difficulties: Indirect Effects

The parents who took part in our online survey reported poor mental health during the early stages of the pandemic, with just over half of parents scoring 3 or more on the GHQ, a cut-off with high specificity and sensitivity commonly used to indicate the presence of a clinically relevant symptoms across diverse samples [49]. Our results highlighted the impact of parental distress as a mediator of the negative impact of the COVID-19 pandemic on child adjustment. This is consistent with a recent American study that showed experiencing the disease in the family as well as the succeeding economic effects both impact family wellbeing [8]. Social learning accounts would suggest the onset and maintenance of child difficulties result from children modeling their parents’ behaviors or ineffective coping strategies. Alternatively, this indirect effect may be carried by changes in parent–child talk, such as inappropriate health risk communication, or compromised parenting practices, such as reduced sensitivity (e.g., [50]). Interestingly, an American study found parents’ worries about COVID-19 were not associated with harsh discipline or reduced warmth [22]. However, this null association may reflect the focus on worrying about contracting and passing on the virus, which may have instead manifested in other behaviors, such as increased handwashing. Future longitudinal research will help tease apart these competing explanations as well as test whether changes in parents’ wellbeing across the pandemic contribute to individual differences in the trajectories of children’s psychosocial problems. That said, it seems evident that family-based interventions may prove most effective in mitigating the negative impact of the pandemic on child adjustment. Furthermore, the advent of innovative work evaluating the success of online family therapy suggests that these interventions do not have to wait until families can meet their therapist in person [51].

Our findings draw on data collected from families across six study sites with different virus infection rates and government approaches, leading to contrasting social, economic, and cultural effects. Reflecting these contrasts, our findings showed site contrasts in levels of COVID-19-related social disruption and stringency. Specifically, families in the USA and UK experienced more disruption than families in all other sites, and Swedish families experienced the least disruption. Nevertheless, with regard to mechanisms of influence on child adjustment, the findings from the different sites indicate striking similarities. This generality of results strengthens the evidence suggesting the cascading impact of economic pressure on child developmental outcomes via parent wellbeing across a variety of family structures, ethnic backgrounds, and geographic locations [52].

## 5. Conclusions

Our study drew on an international sample of 2516 parents and applied tests of measurement invariance to both our outcome and mediating variables to provide meaningful cross-cultural comparisons. That said, our findings should be interpreted considering a number of methodological limitations.

First, our convenience sample of parents from middle to high socioeconomic backgrounds impacts the external validity and generalizability of our findings. The subsamples were restricted in terms of geographic (e.g., American parents in Pennsylvania and Ohio and Chinese parents in Beijing) and socioeconomic (e.g., almost 60% of Italian parents in our study had a tertiary education qualification compared to the national average level of 20% [53]) position and were ethnically homogenous. Here, it is worth noting that, unfortunately, a large proportion of parents in China did not report on their ethnicity, which further limits our understanding of the representativeness of the Chinese subsample. Furthermore, the necessary reliance on online research designs during the pandemic increases the likelihood of selection bias and collider bias [54]. For example, participation is likely associated with increased engagement with social media campaigns, scientific interest, and higher education levels [54]. As a result, we cannot rule out that our models replicate across different countries because those who took part were largely similar (i.e., middle to high socioeconomic backgrounds). However, given we found associations between COVID-19 disruption, family risk factors, and child adjustment within a restricted range of SES, it is likely our estimates are conservative. Further research is required with more diverse samples as well as with families living in low or middle income countries to test whether our results replicate beyond this specific demographic.

Second, the cross-sectional design limits our appreciation of the nature of the mediation findings. The findings may not be pandemic specific given we cannot rule out the impact of pre-existing levels of distress on parents’ current mental health. That said, our sample did not have a history of psychopathology, and our theoretically informed analyses focused on disruption to life due to COVID-19-specific changes in association with current levels of distress [13]. Future longitudinal research is required to rule out alternative explanations regarding the directionality of our results (i.e., child difficulties eliciting parental stress).

Third, like other colleagues, we relied upon a single informant to report on both parents and children, which may inflate associations between constructs. However, as illustrated in Table 2, we found modest associations between family processes and child outcomes. Our results are strengthened by including an objective assessment of COVID-19 experiences. Specifically, a publicly available data set that assessed the stringency of national restrictions enabled us to test the impact of this distal influence on child adjustment. As this measure is strongly positively correlated with national rates of infection [55], it also provides a proxy for national infection rates and a time-specific index of the severity of the disease’s impact. As we move towards more open science, future collaborative efforts that pool individual participant data measuring similar constructs will be helpful in developing an understanding of the varied impact of the pandemic on parent and child adjustment across diverse contexts.

With these caveats in mind, the findings from this international study provide further evidence that the disruptive impact of the COVID-19 pandemic is multigenerational, impacting parents’ wellbeing and children’s social skills and adjustment difficulties. As a result, family-based interventions may prove most effective in mitigating the negative impact of the pandemic on child adjustment.

## Figures and Tables

**Table 1:** Family demographics by site.

	Overall	Australia *n* = 161	China *n* = 336	Italy *n* = 244	Sweden *n* = 795	UK *n* = 706	USA *n* = 274	Between-Site Differences Effect Size
Parent								
Age *M* (*SD*)	37.15 (5.93)	38.89 (5.55)	34.98 (5.75)	39.32 (4.78)	36.63 (4.85)	37.43 (6.92)	37.70 (6.48)	η^2^ = 0.04
Education % degree	63.1%	73.9%	19% ^a^	59.4%	69.8%	67.4%	83.2%	Cramer’s V = 0.11
Child								
Age *M* (*SD*)	5.77 (1.10)	6.09 (1.08)	5.34 (0.99)	5.83 (1.13)	5.65 (1.11)	6.06 (1.05)	5.68 (1.08)	η^2^ = 0.05
Gender % female	47.9%	50.3%	48.2%	51.2%	47.9%	46%	47.8%	Cramer’s V = 0.04
Family								
No. of children *M* (*SD*)	2.02 (1.01)	2.09 (0.78)	1.70 (1.73)	1.72 (0.74)	2.22 (0.84)	2.04 (0.85)	2.00 (0.81)	η^2^ = 0.04

*Note*. degree = undergraduate degree or higher and/or equivalent vocational qualification. ^a^ = 75% missing.

**Table 2:** Robust maximum likelihood estimates for correlations between main study measures.

		1.	2.	3.	4.	5.	6.	7.	8.	9.	10.	11.
1.	Hyperactivity	-										
2.	Conduct Problems	0.86	-									
3.	Emotion Problems	0.54	0.68	-								
4.	Peer Problems	0.50	0.63	0.51	-							
5.	Parent Distress	0.37	0.38	0.39	0.21	-						
6.	Parent–Child Conflict	0.31	0.38	0.25	0.18	0.24	-					
7.	Parent Conflict	0.07	0.16	0.14	0.09	0.09	0.05	-				
8.	Household Chaos	0.44	0.49	0.39	0.29	0.30	0.29	0.17	-			
9.	COVID-19 Disruption	0.18	0.23	0.20	0.22	0.27	0.15	0.11	0.16	-		
10.	Gov. Stringency	0.03	0.07	0.04	0.22	−0.05	0.01	0.10	−0.08	0.03	-	
11.	Socioeconomic Status	−0.18	−0.16	−0.14	−0.08	−0.09	0.06	−0.10	−0.10	−0.06	0.08	-

**Table 3:** Robust Maximum Likelihood Estimates for Structural Equation Model.

		Hyperactivity			Conduct Problems			Emotion Problems			Peer Problems		
		Est	SE	Std Est.	Est	SE	Std Est.	Est	SE	Std Est.	Est	SE	Std Est.
Family Processes	Parent Distress	0.04	0.01	0.18 *	0.03	0.01	0.15 *	0.05	0.01	0.23 *	0.01	0.01	0.04
	Parent-Child Conflict	−0.16	0.02	−0.16 *	−0.21	0.02	−0.22 *	−0.10	0.02	−0.11 *	−0.07	0.02	−0.07
	Couple Conflict	−0.06	0.05	−0.03	0.13	0.05	0.06 *	0.11	0.05	0.06 *	−0.04	0.06	0.02
	Household Chaos	0.08	0.01	0.30 *	0.09	0.01	0.34 *	0.06	0.01	0.24 *	0.06	0.01	0.22 *
COVID-19	Disruption	0.02	0.02	0.04	0.04	0.02	0.07 *	0.03	0.02	0.05	0.08	0.02	0.14 *
	Stringency	0.01	0.00	0.09 *	0.01	0.01	0.12 *	0.01	0.01	0.09 *	0.02	0.01	0.18 *
Background	SES	−0.18	0.03	−0.13 *	−0.13	0.03	−0.09 *	−0.11	0.03	−0.08 *	−0.07	0.03	−0.05
	Child Gender	0.19	0.04	0.11 *	0.14	0.02	0.08 *	−0.01	0.04	−0.00	0.19	0.04	0.11 *
	Child Age	0.02	0.02	0.02	−0.02	0.03	−0.02	0.06	0.02	0.08 *	−0.01	0.02	−0.01
	*R* ^2^	0.29			0.36			0.24			0.16		

*Note*. * *p* < 0.001.

## Data Availability

The data presented in this study are available on request from the corresponding author.
